# Supplementary material for: COVID-19 Vaccination among Czech Dentists
Source: Vaccines (Basel). 2022 Mar 11;10(3):428. doi: 10.3390/vaccines10030428 (PMC8953980; doi:10.3390/vaccines10030428)
Supplement: Supplementary file 1 [file vaccines-10-00428-s001.zip › vaccines-1591757-supplementary.pdf]

vaccines

Table S1. Questions and their classification.

Question number	Question	Question type	Number of Closed-Ended Answer Options	Answer choice
Q1	What is your status/approach to vaccination against COVID-19?	Closed	3	Single
Q2	What was the indication for COVID-19 vaccination?	Semi-closed	3	Single
Q3	Why were you not vaccinated against COVID-19?	Semi-closed	6	Single
Q4	Has your COVID-19 vaccination been completed yet?	Semi-closed	4	Single
Q5	In which month was the COVID-19 vaccination completed?	Closed	9	Single
Q6	What vaccine was used for the vaccination?	Semi-closed	5	Single
Q7	How was vaccination managed?	Semi-closed	4	Single
Q8	Sex	Closed	2	Single
Q9	Age	Closed	6	Single

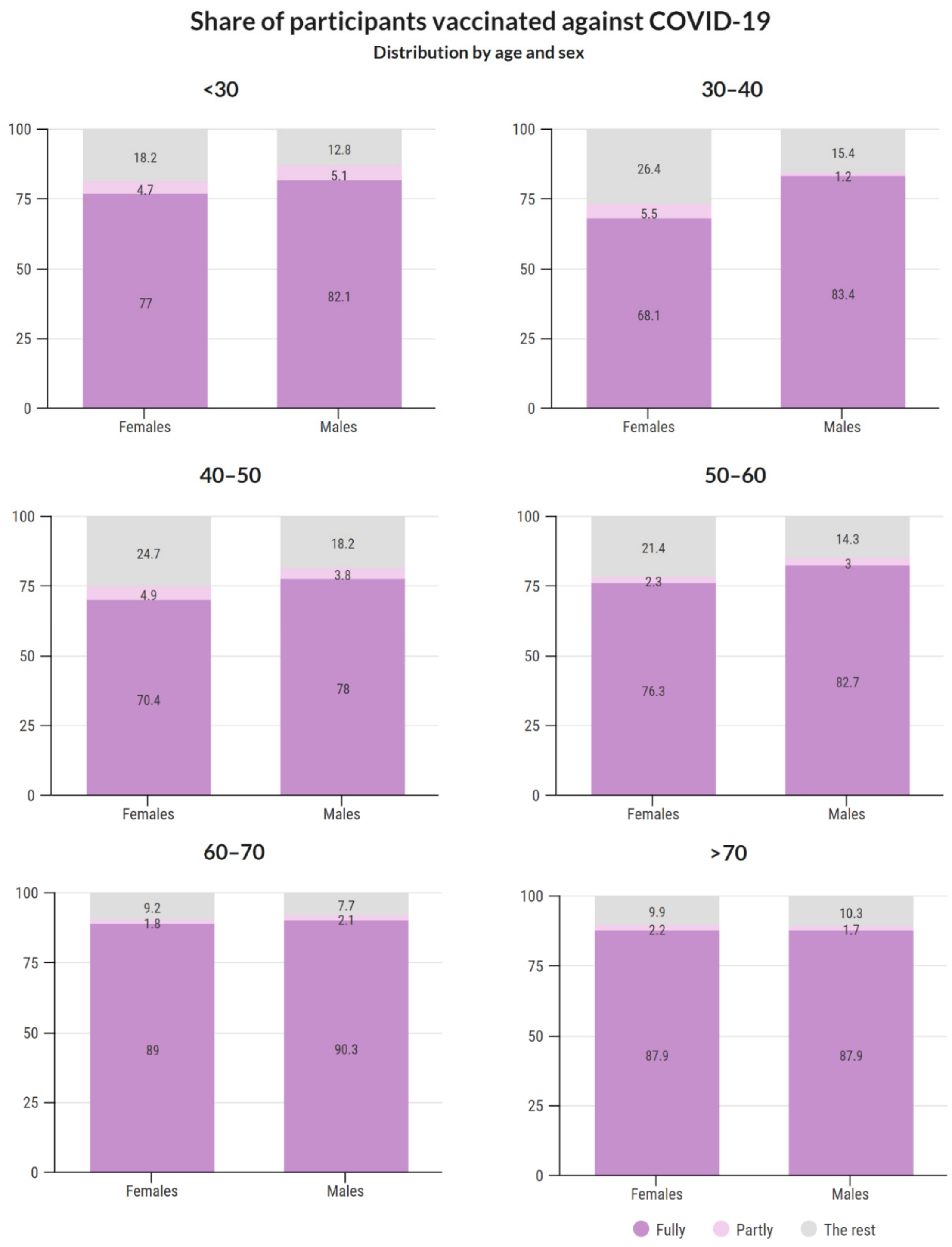

Figure S1. Share of participants vaccinated against COVID-19 based on age and sex. Values are given as percentages.

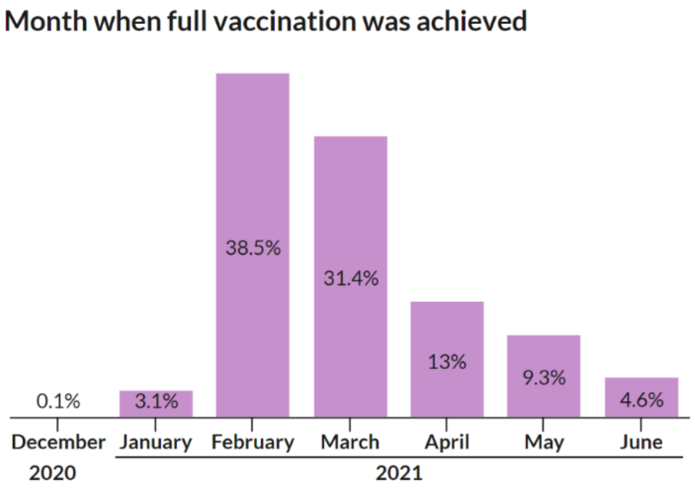

Month	No. of responses	%
December 2020	2	0.1
January 2021	66	3.1
February 2021	832	38.5
March 2021	679	31.4
April 2021	280	13.0
May 2021	201	9.3
June 2021	100	4.6

Figure S2. The month when full vaccination was achieved.
